# Differential organ responses to fumonisins in rabbits: kidney, liver, and spleen membrane fatty acid composition, oxidation markers, and histopathology

**DOI:** 10.3389/fvets.2025.1599805

**Published:** 2025-06-10

**Authors:** Omeralfaroug Ali, Edward Agyarko, Zsolt Gerencsér, Krisztián Balogh, Miklós Mézes, Melinda Kovács, Mohamed Maki, Noureddine Besselma, Haruna Gado Yakubu, András Szabó

**Affiliations:** ^1^Agrobiotechnology and Precision Breeding for Food Security National Laboratory, Department of Animal Physiology and Health, Institute of Physiology and Animal Nutrition, Hungarian University of Agriculture and Life Sciences, Kaposvár, Hungary; ^2^Institute of Agricultural and Food Economics, Hungarian University of Agriculture and Life Sciences, Kaposvár, Hungary; ^3^Department of Feed Safety, Institute of Physiology and Nutrition, Hungarian University of Agriculture and Life Sciences, Gödöllő, Hungary; ^4^HUN-REN-MATE Mycotoxins in the Food Chain Research Group, Hungarian University of Agriculture and Life Sciences, Kaposvár, Hungary; ^5^Department of Biochemistry and Medical Chemistry, Medical School, University of Pécs, Pécs, Hungary; ^6^Agri-Food and Environmental Microbiology Platform (PiMiAA), Department of Molecular and Translational Medicine, University of Brescia, Brescia, Italy; ^7^Department of Animal Science, School of Agriculture, College of Agriculture and Natural Sciences, University of Cape Coast, PMB UCC Cape Coast, Cape Coast, Ghana

**Keywords:** rabbit, fumonisin mycotoxin, fatty acid, oxidative stress, histopathology, clinical chemistry, phospholipids

## Abstract

The study assessed the kidney, liver, and spleen of adult male rabbits (*n* = 10/group) in relation to fumonisin B series exposure (10 and 20 mg FB_1_ + FB_2_ + FB_3_/kg feed) over a period of 65 days. The rabbit growth and feed intake remained unaffected; meanwhile, kidney and liver weights increased. The highest dose provided greater alterations in total phospholipid fatty acid profiles, particularly in the kidney (C20:5n3 and C18:0) and spleen (C18:1n7, C22:0, C20:4n6, and C20:5n3) than in the liver. Neither the kidneys nor the spleens demonstrated modifications in their antioxidant (glutathione and glutathione peroxidase) and lipid peroxidation (malondialdehyde) markers; however, there was a marked drop in the liver glutathione concentration and glutathione peroxidase of the group that administered 20 mg FBs/kg diet, while liver malondialdehyde levels remained unchanged. Serum clinical measures revealed elevated creatinine, total cholesterol, high-density lipoproteins, and gamma-glutamyl transferase activity at the highest FBs dose. Histological scores revealed mild nephrotoxicity and hepatotoxicity in the 20 mg FBs/kg group, accompanied by a mild to moderate lesion score in the spleen. Overall, FBs exposure elicited diverse organ-specific adverse effects, with severity increasing at higher doses. Despite these alterations, rabbits demonstrated adaptability to FBs over the study period, as indicated by steady growth performance.

## 1 Introduction

Mycotoxins, which are fungal secondary metabolites, are prevalent in nature and adversely affect the environment and its biocomponents: humans, animals, and plants. The recent analytical advancements and academic studies illustrate their high risk due to the identification of novel structures/isomers, as well as global climate change, which is likely to increase fungal activities. For example, throughout 2024, the Dutch State Mines (DSM)-Firmenich reported that 98% of 1,271 analyzed samples from 38 countries tested positive for at minimum 10 mycotoxins and metabolites (1). Among these mycotoxins, the fumonisins (FUM), mostly produced by *Fusarium verticillioides* and *Fusarium proliferatum*, ranked as the second most often occurring mycotoxins in completed feed (exceeding 60%), especially in regions characterized by high temperatures and humid climates. Thus, this mycotoxin poses a severe threat to the general health by getting access to the food/feed chain, particularly through corn, its main targeted crop. Up to date, numerous structures of FUMs have been detected and identified, which have been collectively categorized into diverse series: e.g., A, B, C, and P (2). The B series, containing FB_1_, FB_2_, FB_3_, and more, is recognized as the most produced FUM series by *Fusarium verticillioides*, with FB1 reaching 70% of the total production (3). Hence, it makes sense to investigate the B series exposure consequences on livestock animals. Nonetheless, legislations yet refer only to the presence of FB_1_, FB_2_, and FB_3_ in animal feed, varying across regions, with FB_1_ + FB_2_ regulated in the European Union and FB_1_ + FB_2_ + FB_3_ in the USA (4, 5). However, among FB toxins, FB_1_ has received great attention due to its high production rate by fungi and high toxicity level (6).

Generally, FB series exert a range of health implications (ranging from metabolic disturbances to cancer), which can vary substantially due to factors like the dose, exposure period, species, and organ (6). In rabbits, FB_1_-toxic effects often appear subclinical; yet, clinical indications featuring weight loss, anorexia, reproductive abnormalities, and increased susceptibility to infections have been reported (7). Furthermore, this toxin is majorly acknowledged as nephrotoxic, hepatotoxic, and neurotoxic in rabbits (8–11) and has been reported to disrupt the metabolism of hematopoietic organs (12, 13). Histological data confirmed centrilobular lipid infiltration and cellular necrosis in the rabbit hepatic tissue, in conjunction with nephrosis in the proximal tubules of kidneys, while *in vitro* studies highlighted that FB_1_ exhibited both cytotoxic and genotoxic effects on rabbit kidney cells (14). Hence, rabbit kidneys are likely the primary target organs for FB_1_. However, its precise impacts on rabbit organ functionality, membrane lipids, potential oxidative stress, and clinical parameters are not well-established, especially when co-exposed with other structures within the FB series. These mycotoxins, which are structurally resembling sphingoid bases, disrupt the synthesis of ceramides through the inhibition of ceramide synthase, ultimately leading to disturbance of the lipid composition of cell membranes (6, 10). Nonetheless, the literature on membrane lipids is concentrated majorly on rats and swine as animal models, while rabbits remain slightly explored. In rodents, prominent distortions in membrane lipid composition observed include increases in cholesterol, phosphatidylethanolamine, and C20:4n6-PE/PC, but decreases in sphingomyelin, long and very long polyunsaturated fatty acids (especially n3 fatty acids), and overall polyunsaturated ratios to saturation and monounsaturation (6, 15). These modifications are likely to affect membrane integrity and cellular signaling (16, 17). With relation to the redox system, the red blood cell hemolysate, spermium, and testis of rabbits remained not affected upon 65 and 10 days of oral exposure to FB_1_ (18–20), highlighting a valuable question on rabbit potential resistance to FB mycotoxins.

Despite the inclusion of corn being less relevant to the finished diets of rabbits and these animals being at a low risk level, the literature lacks empirical data on FB_1_ + FB_2_ + FB_3_ toxicity levels in their organs. Thus, this study aims to address this gap by investigating the *in vivo* effects of FB_1_ + FB_2_ + FB_3_ exposure on adult male rabbits. Specifically, the study assessed the impact of these mycotoxins on rabbit organs, including the kidney, liver, and spleen, using membrane lipid composition, antioxidant and lipid peroxidation biomarkers, clinical biochemical parameters, and histopathological lesions as endpoints. The findings of this study contribute to the broader field of mycotoxin research and provide valuable insights into the potential risks associated with FUM exposure in rabbits.

## 2 Materials and methods

### 2.1 Mycotoxin production and analysis

The *Fusarium verticillioides* (MRC 826) fungal strain was used for FBs production, as described in detail (21). The final concentrations of FB_1_ in the air-dried culture material from different batches ranged between 2,000 and 4,000 mg/kg. The FB_2_ and FB_3_ concentrations in the inoculum materials were approximately 30% and 10–15% of the FB_1_ content, respectively. The diet of the control group was free of detectable quantities of FBs, while diets of the experimental groups were supplemented by the fungal culture so as to provide 10 and 20 mg FBs/kg diet. All diets were confirmed to be free of deoxynivalenol (DON), zearalenone (ZEN), and T-2 toxin, whereby the analyzed feeds had concentrations below the detection limit (0.053, 0.005, and 0.011 mg/kg for DON, ZEN, and T-2 toxin, respectively). The concentration of FBs was quantified with an LC–MS-2020 mass spectrometer (Shimadzu, Kyoto, Japan).

### 2.2 Ethical allowance

All investigations were conducted in accordance with the Hungarian Animal Protection Act (40/2013. (II. 14.)), in line with the EU Directive 2010/63 for the protection of animals used for scientific purposes (22). The allowance reference for the investigations was SOI/31/00308–10/2017 (KA2114), with an approval date of 27 March 2017. Methods were carried out in accordance with relevant guidelines and regulations that are reported in accordance with ARRIVE (Animal Research: Reporting of *in vivo* Experiments) guidelines.^1^

### 2.3 Animals, experimental site, and design

Total of 30 healthy adult Pannon White rabbit bucks of the same age (24 weeks) and relatively similar body weights were housed at the breeding farm of Kaposvár Campus, the Hungarian University of Agriculture and Life Sciences, in which the experiment was carried out at the experimental animal farm of the same institution. The site environmental temperature was adjusted to 24°C, and the photoperiod was natural (during October and November), and the light period ranged between 10 and 12 h. The experimental animals were randomly assigned to individual pens (40 × 98 × 57 cm; width, length and height, respectively), each 10 animals represented a group. After a 14-day adaptation period, 10 rabbits received a control diet (feed free from mycotoxins and medications), whereas rabbits from other groups were separately fed FBs (FB_1 +_ FB_2 +_ FB_3_)-contaminated diets with doses (10 and 20 mg/kg feeds) above the EU recommended limit in diets intended for rabbit feeding (4). These different diets and drinking water were offered *ad libitum* for 65 days. The fatty acid profile and chemical composition of the experimental diets are shown in Table 1. Throughout the treatment, both body weight and feed intake were recorded on an individual basis, as per Szabó et al. (19). Based on these measurements, body weight gains and feed conversion efficiencies were calculated.

**Table 1:** The chemical and fatty acid composition of the experimental diet.

Chemical composition	Value
Dry material (%)	89
Crude protein (%)	14.53
Ether extract (%)	2.4
Crude fibre (%)	17.08
Ash (%)	7.51
Lysine (%)	0.9
Methionine (%)	0.41
Calcium (%)	0.88
Phosphorus (%)	0.52
Sodium (%)	0.19
vitamin A (IU/kg)	14,000
vitamin D3 (IU/kg)	1,300
vitamin E (mg/kg)	107
Digestible energy (MJ/kg)	9.7

Fatty acid (diet)	Weight % of total FAME
C12:0	0.047
C14:0	0.179
C15:0	0.136
C16:0	14.301
C16:1n7	0.197
C17:0	0.124
C18:0	2.73
C18:1n9	36.781
C18:1n7	0.837
C18:2n6	38.633
C18:3n3	3.834
C20:0	0.421
C20:1n9	0.495
C20:2n6	0.044
C21:0	0.036
C20:4n6	0.068
C22:0	0.609
C24:0	0.449
C22:6n3	0.078

The feed was withdrawn 12 h before sacrifice, leaving only drinking water accessible. At the end of this period, the rabbits were anesthetised with euthanyl-pentobarbital sodium (400 mg/mL, Dechra Veterinary Products, Shrewsbury, UK). Euthanasia was performed through exsanguination. Liver, kidney, and spleen were weighed, sampled, and preserved at a temperature of −70°C for further analysis (refer to subsequent sections). Blood samples were collected in heparinized Vacutainer tubes (Fisher Scientific, Bishop Meadow Road, Loughborough, Leicestershire, UK).

### 2.4 Lipid analysis

Liver, kidney and spleen samples, ca. 100 mg wet weight per sample, were thawed at room temperature, then homogenized with a 20-fold mixture of chloroform and methanol (2:1 volume ratio) and an IKA Ultra Turrax, T18 (IKA, Staufen, Germany). The total lipid content was then extracted as described by Folch et al. (23). Solvents of high purity (99.5% or higher, sourced from Merck, Schnelldorf, Germany) were used, whereas 0.01% *w/v* butylated hydroxytoluene was added to prevent fatty acid oxidation during the analysis.

For separation of lipid fractions, the extracted total lipids were transferred into glass chromatographic columns filled with 150 mg of silica gel (200–425 mesh, Merck #236772) for every 5 mg of lipids, as described by Leray et al. (24). Neutral lipids were eluted through the addition of 5 mL chloroform, followed by the addition of 7.5 mL acetone–methanol (9:1 volume ratio) for the given fat quantity. Total phospholipids were then eluted with 5 mL pure methanol. This final fraction was evaporated under a stream of nitrogen and trans-methylated by Christie’s base-catalyzed NaOCH_3_ method (25).

Fatty acid methyl esters (FAME) were afterwards extracted by 300 μL of ultrapure n-hexane. Gas chromatography was executed via an AOC 20i automatic injector connected to a Shimadzu Nexis 2030 system (Kyoto, Japan), which was equipped with a Phenomenex Zebron ZB-WAXplus capillary GC column (30 m × 0.25 mm ID, 0.25 μm film, Phenomenex Inc., Torrance, CA, USA) and a flame ionization detector (FID). The operating conditions included an injector temperature of 220°C, a detector temperature of 250°C, and a helium flow rate of 28 cm/s. The oven temperature was programmed to start at 60°C with a 2 min hold, increase to 150°C, increase from 150 to 180°C at a rate of 2°C/min with a 10 min hold at 180°C, and finally increase from 180 to 220°C at a rate of 2°C/min with a 16 min hold at 220°C. Nitrogen was used as the makeup gas. Data analysis was performed via LabSolutions 5.93 software, utilizing the PostRun module (Shimadzu, Kyoto, Japan) with manual peak integration. Fatty acids were identified on the basis of retention times of an external CRM standard (Supelco 37 Component FAME Mix, Merck-Sigma Aldrich, CRM47885). The C22:4 n6 and C22:5 n6 standards were purchased from Merck (cat. no.: D3534) and Larodan (Solna, Sweden, cat. no.: 10–2,265-4), respectively. The results for fatty acids are presented as the percentage of total FAMEs by weight.

### 2.5 Measurement of the antioxidant and oxidative markers

Firstly, samples were thawed to room temperature and then mixed in a saline solution (0.65% (*w/v*) NaCl) at a ratio of 1:9 for the purpose of biochemical evaluation. The malondialdehyde (MDA) concentration was determined in the original homogenates of 1:9 ratio, whereas the indicators of the glutathione redox system were evaluated via the supernatant obtained post-centrifugation of the homogenates (10,000 × g, 3 min, 4°C), which is equivalent to the microsomal fraction. The concentration of MDA was established through the formation of a complex with 2-thiobarbituric acid in an acidic environment at elevated temperature (Sigma, St. Louis, MO, USA) following the method proposed by Fawaeir et al. (26). Subsequently, 10% (*w/v*) trichloroacetic acid (Carlo Erba, Rodano, Italy) was used to modify the acidic environment. The reaction duration was 20 min at 100°C. After cooling and centrifugation (2,500 rpm, 4°C), the absorbance was measured from the supernatant against the reagent blank at a wavelength of 535 nm (27).

The concentration of reduced glutathione in the samples was determined accordingly with the method described by Sedlak and Lindsay (28). This method relies on the color complex formation of the free SH group of glutathione with a compound that is reactive to sulfhydryl groups. The protein content of the samples was precipitated with 10% (*w/v*) trichloroacetic acid (Carlo Erba, Rodano, Italy), and measurements were taken from the supernatant fraction after centrifugation (10,000 × g, 3 min, 4°C). The concentration of GSH can be ascertained by measuring the absorbance at 412 nm.

The principle of determining GPx activity involves the oxidation of GSH to glutathione disulfide by GPx in the presence of ROS (29). After incubation for 10 min at room temperature (25 ± 2°C), the reaction was halted by precipitating the protein with 10% (*w/v*) trichloroacetic acid (Carlo Erba, Rodano, Italy). The reduction in the quantity of GSH was ascertained by measuring the absorbance of the complex formed with 5,5-dithiobis-(2-nitrobenzoic acid) (Sigma, St. Louis, MO, USA) at a wavelength of 412 nm. Enzyme activity is expressed in units of 1 nmol GSH oxidation per minute in the system used at 25°C.

The GSH content and the GPx activity were determined in relation to the protein content of the supernatant fraction using the Folin–Ciocalteu phenol reagent (30).

### 2.6 Serum biochemical analysis

**Figure 1:**
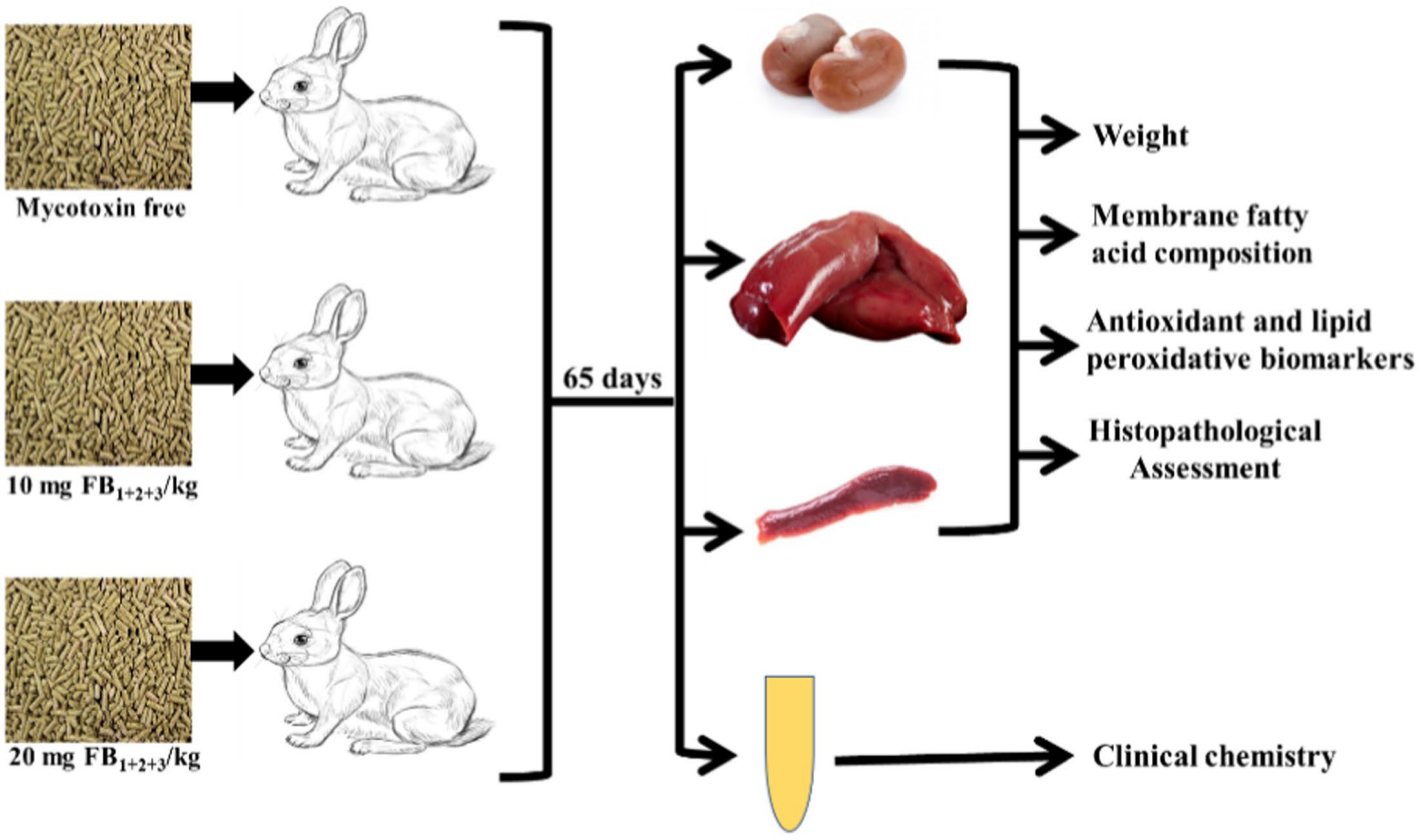
Chart represents the experimental design and analytical approaches performed.

Serum was immediately extracted from clotted blood samples (see Figure 1) via centrifugation for 10 min at 1,000 × g (SIGMA 3-30KS refrigerated centrifuge, Osterode am Harz, Germany). A variety of clinical parameters, including serum nitrogenous compounds, lipid metabolites, enzyme activities, and ion levels, were analyzed. This analysis was conducted in a veterinary laboratory (Vet-Med Laboratory Ltd., Budapest, Hungary). The Roche Hitachi 917 Chemistry Analyzer (Hitachi, Tokyo, Japan), was utilized for this purpose along with commercial diagnostic kits supplied by Diagnosticum Ltd., Budapest, Hungary.

### 2.7 Histopathological preparation and assessment

The tissue samples were preserved in a 10% neutrally buffered formalin before being embedded into paraffin. Slides of five micrometers were prepared for light microscopic examination and were stained with hematoxylin-eosin. The primary pathological changes were identified and rated on the basis of their scope and intensity as follows (31): 0 = no change, 1 = minor/limited/few, 2 = moderate/moderate scale/moderate quantity, and 3 = severe/widespread/many. The histopathological examination was carried out in compliance with Act #2011 (03.30) issued by the Hungarian Ministry of Agriculture and Rural Development and adhered to the ethical guidelines of the OECD Good Laboratory Practice for Chemicals.

### 2.8 Data analysis

The evaluation of group means (including enzyme activity, initial and final body weight, and fatty acid profile data within individual rows) was performed via univariate analysis of variance (ANOVA), whereas the Tukey “*post hoc*” test was employed to identify intergroup differences. The Spearman’s rho rank correlation method was performed between ranked administered FBs’ levels and the obtained continuous dataset of various parameters, whereas Pearson’s correlation test was employed between the various continuous datasets to identify potential interrelationships. The significance level for all tests was set at *p*-value ≤ 0.05. The evaluation was performed by IBM SPSS 29 for Windows (2022).

Sparse Partial Least Squares Classification (Discriminant Analysis, sPLS-DA) was performed for dimension reduction and variable selection for classification with the highest accuracy (32). Additionally, two-way cluster analysis was carried out to achieve possible natural grouping, but only as an explorative tool. Both test types were performed via R project version 4.1.2 (2017) and the mixOmics package (6.18.1.) (33).

## 3 Results

### 3.1 Animal performance and organ weight

Results on the animal performance can be seen in Table 2. The results showed that live body weight, both initial and final, remained unaffected across all experimental groups. Similarly, there were no significant changes observed in the spleen weight and feed intake among the different treatment groups. However, notable alterations were observed in the liver and kidney weights. In animals exposed to the highest level of FBs (20 mg/kg diet), there was a significant increase in the weights of both the liver and kidneys when compared to the group fed on 10 mg FBs/kg feed.

**Table 2:** Weights of animals and organs and feed intake of experimental rabbits (*n* = 10/group) during the whole trial period; 65 days.

Parameter	Control	10 mg FBs/kg diet	20 mg FBs/kg diet
	Mean ± SD	Mean ± SD	Mean ± SD
Initial body weight (g)	4,380 ± 345	4,387 ± 335	4,390 ± 325
Final body weight (g)	4,819 ± 350	4,686 ± 309	4,619 ± 486
Liver (g)	91.7 ± 17.6ab	96.9 ± 13.2a	80.9 ± 11.6b
Kidney (g)	19.8 ± 1.92ab	20.8 ± 1.95a	18.4 ± 1.77b
Spleen (g)	1.80 ± 0.41	1.58 ± 0.47	1.62 ± 0.30
Cumulative feed intake (g)	11931.3 ± 1309.2	11555.7 ± 639.5	10949.7 ± 1221.1

Data represent mean ± standard deviation (SD). a and b letters indicate intergroup differences with *p*-value < 0.05.

### 3.2 Kidney total phospholipid fatty acid profile

The analysis of the kidney total phospholipid fatty acid composition revealed specific alterations in fatty acid composition (see Table 3). There were significant decreases in the proportions of C14:0 (myristic acid) and C18:1n7 (vaccenic acid) in the kidneys after exposure to 20 mg FBs/kg, while an increase was observed in C22:0 (behenic acid) within the same group. However, none of their calculated indices, namely total saturation and total unsaturation, showed significant alterations. Among the polyunsaturated fatty acids, only the omega-3 (n3) fatty acids were notably affected. Animals exposed to the highest dose of FBs revealed a proportional elevation (approximately ¾ fold increase) in C20:5n3 (eicosapentaenoic acid, EPA), while there was a proportional depletion in C22:6n3 (docosahexaenoic acid, DHA). The extent of EPA elevation was more pronounced (showing a correlation (*r* = 0.702) with FBs dose), leading to an overall increase in total n3 fatty acids. This increase in n3 fatty acids co-occurred with a decrease in the omega-6 to omega-3 ratio (n6:n3).

**Table 3:** The fatty acid composition of kidney total phospholipids from rabbits (*n* = 10/group).

Fatty acid	Control	10 mg FBs/kg diet	20 mg FBs/kg diet
	Mean ± SD	Mean ± SD	Mean ± SD
C14:0	0.10 ± 0.03a	0.10 ± 0.01ab	0.08 ± 0.01b
C16:0	15.8 ± 1.56	15.7 ± 1.44	14.8 ± 0.55
C16:1n7	0.35 ± 0.09	0.37 ± 0.11	0.29 ± 0.09
C18:0	18.1 ± 1.83	16.9 ± 0.96	17.4 ± 0.41
C18:1n9	15.4 ± 1.43	16.2 ± 0.94	16.1 ± 0.81
C18:1n7	1.76 ± 0.20ab	1.87 ± 0.23a	1.59 ± 0.19b
C18:2n6	25.3 ± 1.53	25.5 ± 1.36	25.8 ± 0.80
C18:3n6	0.04 ± 0.01	0.04 ± 0.01	0.05 ± 0.01
C18:3n3	0.24 ± 0.06	0.27 ± 0.04	0.27 ± 0.02
C20:0	0.15 ± 0.07	0.12 ± 0.02	0.13 ± 0.05
C20:1n9	0.26 ± 0.06	0.29 ± 0.08	0.28 ± 0.05
C20:2n6	0.61 ± 0.17	0.63 ± 0.20	0.62 ± 0.10
C20:3n6	0.91 ± 0.26	0.91 ± 0.22	1.07 ± 0.18
C20:3n3	0.05 ± 0.02	0.06 ± 0.02	0.05 ± 0.02
C20:4n6	19.1 ± 1.45	19.3 ± 1.35	19.7 ± 0.47
C20:5n3	0.20 ± 0.07b	0.26 ± 0.06b	0.33 ± 0.04a
C22:0	0.02 ± 0.00b	0.02 ± 0.01b	0.02 ± 0.00a
C22:1n9	0.02 ± 0.00	0.01 ± 0.00	0.02 ± 0.01
C22:4n6	0.57 ± 0.08	0.55 ± 0.04	0.54 ± 0.03
C22:5n6	0.41 ± 0.08	0.44 ± 0.07	0.42 ± 0.02
C22:5n3	0.29 ± 0.03	0.29 ± 0.03	0.30 ± 0.03
C22:6n3	0.20 ± 0.05a	0.20 ± 0.04a	0.16 ± 0.03b
C24:0	0.03 ± 0.01	0.02 ± 0.01	0.02 ± 0.01
C24:1n9	0.03 ± 0.02	0.03 ± 0.01	0.03 ± 0.01
Saturation	34.2 ± 3.25	32.8 ± 0.72	32.4 ± 0.55
Monounsaturation	17.8 ± 1.59	18.8 ± 0.88	18.3 ± 0.90
Polyunsaturation	47.9 ± 2.32	48.4 ± 0.78	49.3 ± 0.85
n6	47.0 ± 2.27	47.3 ± 0.74	48.2 ± 0.89
n3	0.98 ± 0.11b	1.08 ± 0.10ab	1.11 ± 0.08a
n6:n3	48.1 ± 4.60a	44.0 ± 4.05ab	43.5 ± 3.56b
Unsaturation index	157.9 ± 8.13	160.4 ± 2.73	162.7 ± 1.75
Average chain length	18.2 ± 0.06	18.2 ± 0.02	18.2 ± 0.02

Data represent mean ± standard deviation (SD). n6, omega-6 fatty acids; n3, omega-3 fatty acids; n6:n3, omega-6 to omega-3 ratio. a and b letters indicate intergroup differences with *p*-value < 0.05.

**Figure 2:**
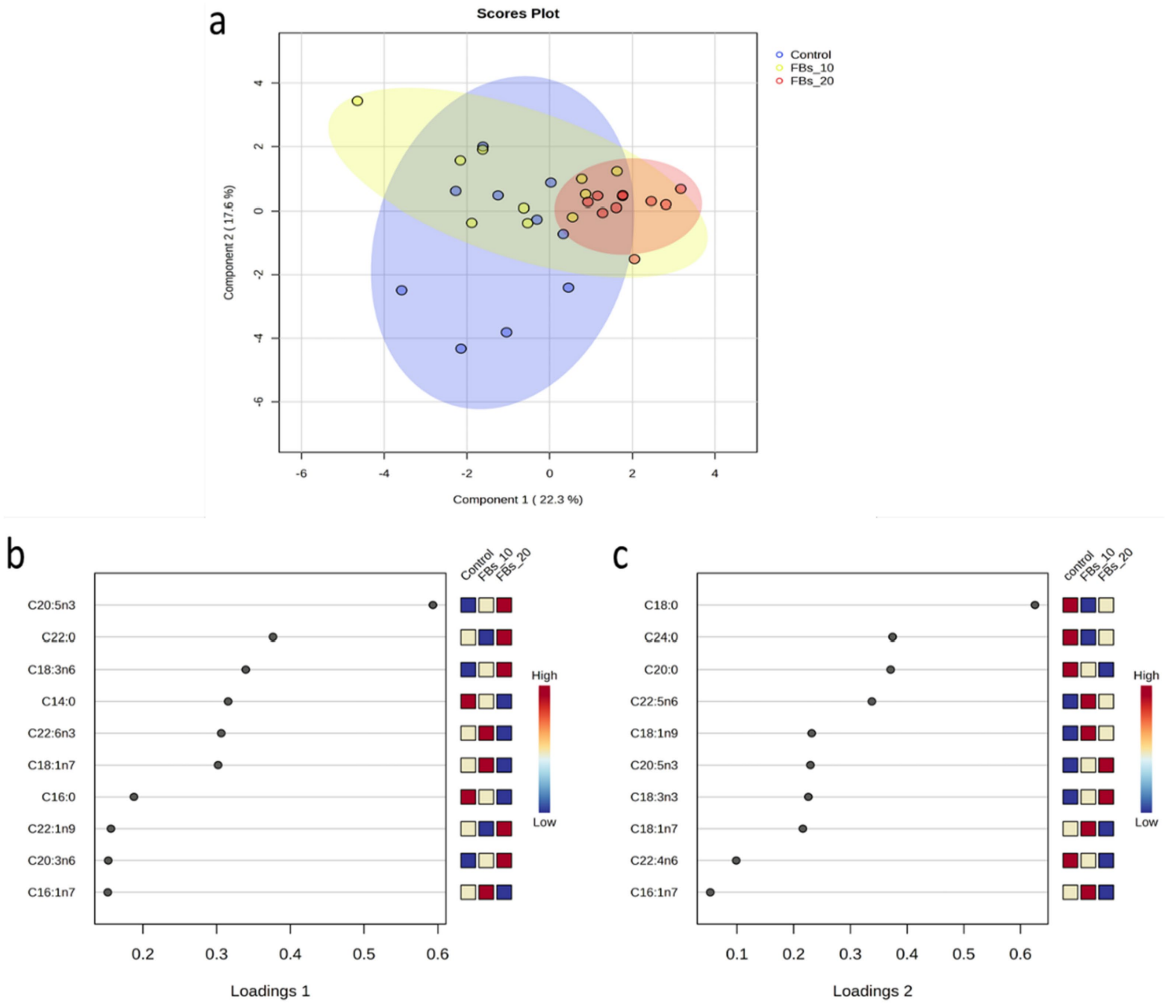
**(a)** The sPLS-DA score plot of the group classification on the basis of the kidney total phospholipid fatty acid composition dataset with ellipses representing 95% confidence intervals. **(b,c)** The first two loadings of the sPLS-DA model of kidney total phospholipid classification.

The principal component analysis (PCA) did not show plain separation of groups along the different component axes. However, the sparse partial least squares classification (sPLS-DA) revealed a clear distinction between the control group and the group fed a 20 mg FBs/kg diet (see Figure 2a), with one renal sample being misclassified through discriminant analysis (*data not shown*). This classification explained 39.1% of the total variance, with EPA and C18:0 (stearic acid) being the major fatty acids contributing to the variation on loadings 1 and 2, respectively (see Figures 2b,c).

### 3.3 Liver total phospholipid fatty acid profile

The liver total phospholipid fatty acid composition can be seen in Table 4. The results from animals treated with FBs display systematic patterns related to the proportions of myristic acid, C16:1n7 (palmitoleic acid), and C20:4n6 (arachidonic acid). However, C22:1n9 (erucic acid) was the only fatty acid that showed a consistent pattern, with a proportional, dose-dependence increase observed in response to exposure to FBs. Further non-systematic patterns were also noticed in the calculated indices, such as total polyunsaturation, total n6 fatty acids, and average chain length. Among all calculated indices, only the unsaturation index showed a significant alteration between the control and the group fed the highest dose of FBs, indicating an increase due to FBs exposure. When principal component analysis (PCA) and sparse partial least squares classification (sPLS-DA) were performed, neither analysis provided a clear separation between the groups.

**Table 4:** The fatty acid composition of liver total phospholipids from rabbits (*n* = 10/group).

Fatty acid	Control	10 mg FBs/kg diet	20 mg FBs/kg diet
	Mean ± SD	Mean ± SD	Mean ± SD
C14:0	0.11 ± 0.01b	0.12 ± 0.02a	0.10 ± 0.02b
C16:0	18.3 ± 0.86	19.3 ± 1.57	18.0 ± 1.11
C16:1n7	0.54 ± 0.15ab	0.58 ± 0.18a	0.40 ± 0.15b
C18:0	26.1 ± 1.33	25.9 ± 2.09	25.4 ± 1.26
C18:1n9	11.6 ± 2.26	12.1 ± 2.69	11.1 ± 2.19
C18:1n7	1.11 ± 0.26	1.08 ± 0.28	0.88 ± 0.19
C18:2n6	28.6 ± 1.90	27.8 ± 1.68	29.8 ± 1.96
C18:3n3	0.73 ± 0.17	0.74 ± 0.16	0.78 ± 0.11
C20:0	0.10 ± 0.01	0.10 ± 0.02	0.09 ± 0.01
C20:1n9	0.29 ± 0.13	0.35 ± 0.19	0.25 ± 0.14
C20:2n6	0.90 ± 0.32	1.04 ± 0.50	0.75 ± 0.38
C20:3n6	0.87 ± 0.28	0.79 ± 0.12	0.73 ± 0.16
C20:3n3	0.06 ± 0.02	0.09 ± 0.04	0.07 ± 0.03
C20:4n6	8.34 ± 1.03ab	7.77 ± 1.21b	9.06 ± 0.87a
C20:5n3	0.05 ± 0.01	0.05 ± 0.02	0.05 ± 0.01
C22:1n9	0.01 ± 0.00b	0.02 ± 0.01a	0.02 ± 0.00a
C22:4n6	0.84 ± 0.18	0.86 ± 0.14	0.98 ± 0.10
C22:5n6	0.85 ± 0.15	0.90 ± 0.13	0.95 ± 0.08
C22:5n3	0.38 ± 0.20	0.29 ± 0.03	0.36 ± 0.04
C22:6n3	0.21 ± 0.03	0.19 ± 0.03	0.21 ± 0.03
C24:0	0.06 ± 0.10	0.03 ± 0.02	0.03 ± 0.01
Saturation	44.7 ± 1.42	45.4 ± 2.88	43.6 ± 0.64
Monounsaturation	13.5 ± 2.67	14.2 ± 2.96	12.6 ± 2.51
Polyunsaturation	41.8 ± 2.24ab	40.4 ± 1.97b	43.7 ± 2.38a
n6	40.3 ± 2.14ab	39.1 ± 1.95b	42.3 ± 2.38a
n3	1.43 ± 0.31	1.35 ± 0.16	1.46 ± 0.14
n6:n3	29.1 ± 5.05	29.2 ± 3.44	29.2 ± 3.31
Unsaturation index	121.8 ± 3.96b	118.4 ± 3.76b	126.6 ± 4.09a
Average chain length	17.9 ± 0.03ab	17.9 ± 0.04b	17.9 ± 0.04a

Data represent mean ± standard deviation (SD). n6, omega-6 fatty acids; n3, omega-3 fatty acids; n6:n3, omega-6 to omega-3 ratio. a and b letters indicate intergroup differences with *p*-value < 0.05.

### 3.4 Spleen total phospholipid fatty acid profile

Table 5 shows the spleen total phospholipid fatty acid profile. Compared to control animals, those fed a diet containing 20 mg FBs/kg showed proportional depletions in C16:0 (palmitic acid), behenic acid, and C24:0 (lignoceric acid). In contrast, the proportion of C20:0 (arachidic acid) increased in animals fed 10 mg FBs/kg feed. Almost all proportions of monounsaturated fatty acids exhibited decreases in animals fed 20 mg FBs/kg feed. This was specifically observed in palmitoleic acid, vaccenic acid, C20:1n9 (gadoleic acid), erucic acid, and C24:1n9 (nervonic acid). Some polyunsaturated fatty acids were also altered by the highest dose of FBs. For instance, both C20:2n6 (eicosadienoic acid) and DHA were proportionally decreased, whereas the proportion of C20:4n6 (arachidonic acid, AA) was significantly increased. When the fatty acid-calculated indices were analyzed, significant alterations were observed only in the group fed 20 mg FBs/kg. The total monounsaturation level showed a decrease, in contrast with observations in total polyunsaturation, total n6 fatty acids, the unsaturation index, and average chain length, all of which showed proportional increases.

**Table 5:** The fatty acid composition of spleen total phospholipids from rabbits (*n* = 10/group).

Fatty acid	Control	10 mg FBs/kg diet	20 mg FBs/kg diet
	Mean ± SD	Mean ± SD	Mean ± SD
C14:0	0.23 ± 0.02	0.22 ± 0.03	0.21 ± 0.04
C16:0	21.8 ± 0.64a	21.8 ± 0.71a	21.0 ± 0.64b
C16:1n7	0.33 ± 0.02a	0.33 ± 0.06a	0.28 ± 0.04b
C18:0	18.9 ± 0.36	19.0 ± 0.46	19.6 ± 0.46
C18:1n9	11.4 ± 0.24	11.3 ± 0.44	11.1 ± 0.40
C18:1n7	2.04 ± 0.09a	1.99 ± 0.13a	1.74 ± 0.12b
C18:2n6	12.5 ± 0.72	12.9 ± 0.35	12.8 ± 1.24
C18:3n6	0.16 ± 0.01	0.17 ± 0.01	0.17 ± 0.01
C18:3n3	0.12 ± 0.01	0.12 ± 0.01	0.15 ± 0.04
C20:0	0.22 ± 0.02b	0.31 ± 0.11a	0.26 ± 0.04ab
C20:1n9	0.61 ± 0.02a	0.65 ± 0.05a	0.55 ± 0.05b
C20:2n6	0.95 ± 0.08a	0.93 ± 0.07a	0.83 ± 0.04b
C20:3n6	1.16 ± 0.04	1.11 ± 0.04	1.13 ± 0.10
C20:3n3	0.05 ± 0.01	0.06 ± 0.01	0.05 ± 0.00
C20:4n6	22.0 ± 0.42b	21.8 ± 0.62b	22.7 ± 0.48a
C20:5n3	0.23 ± 0.02	0.26 ± 0.04	0.24 ± 0.02
C22:0	0.08 ± 0.01a	0.07 ± 0.02b	0.05 ± 0.01c
C22:1n9	0.10 ± 0.01a	0.09 ± 0.02a	0.07 ± 0.01b
C22:4n6	4.06 ± 0.11	3.92 ± 0.13	4.03 ± 0.18
C22:5n6	1.15 ± 0.08	1.18 ± 0.06	1.18 ± 0.04
C22:5n3	1.60 ± 0.07	1.58 ± 0.11	1.63 ± 0.05
C22:6n3	0.11 ± 0.01a	0.10 ± 0.02ab	0.09 ± 0.01b
C24:0	0.08 ± 0.01a	0.08 ± 0.01a	0.06 ± 0.01b
C24:1n9	0.08 ± 0.01a	0.08 ± 0.01a	0.05 ± 0.02b
Saturation	41.3 ± 0.73	41.4 ± 0.62	41.2 ± 0.48
Monounsaturation	14.6 ± 0.30a	14.5 ± 0.55a	13.8 ± 0.55b
Polyunsaturation	44.1 ± 0.74b	44.1 ± 0.66b	45.0 ± 0.84a
n6	42.0 ± 0.73b	42.0 ± 0.53b	42.8 ± 0.86a
n3	2.11 ± 0.08	2.12 ± 0.14	2.16 ± 0.07
n6:n3	19.9 ± 0.75	19.9 ± 1.22	19.8 ± 0.85
Unsaturation index	165.8 ± 2.51b	165.1 ± 2.41b	168.3 ± 1.31a
Average chain length	18.3 ± 0.02b	18.3 ± 0.03ab	18.4 ± 0.02a

Data represent mean ± standard deviation (SD). n6, omega-6 fatty acids; n3, omega-3 fatty acids; n6:n3, omega-6 to omega-3 ratio. a and b letters indicate intergroup differences with *p*-value < 0.05.

**Figure 3:**
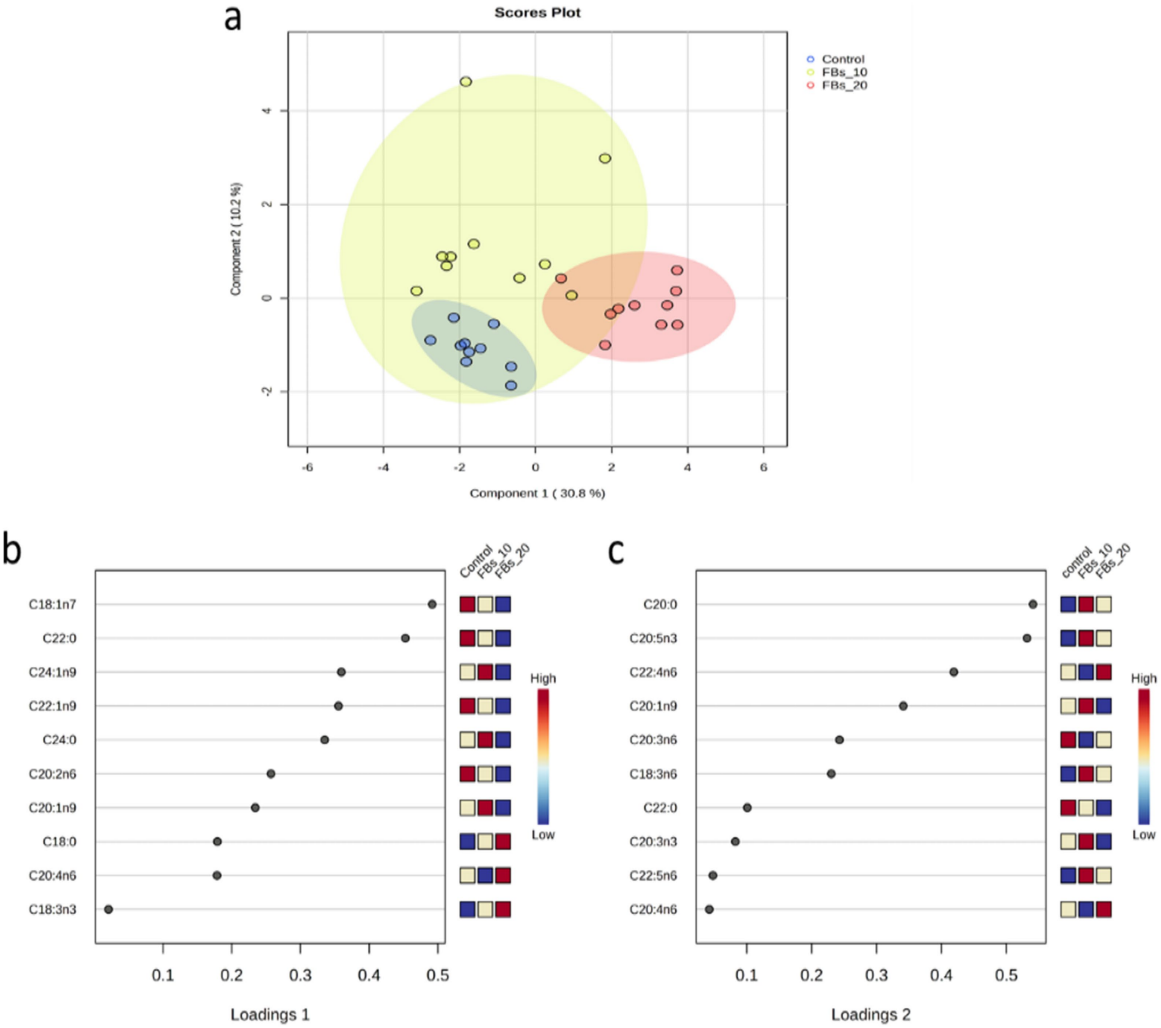
**(a)** The sPLS-DA score plot of the group classification on the basis of the spleen total phospholipid fatty acid composition dataset with ellipses representing 95% confidence intervals. **(b,c)** The first two loadings of the sPLS-DA model of spleen total phospholipid classification.

The sPLS-DA analysis successfully distinguished between the control and the group exposed to 20 mg FBs (see Figure 3a), with all samples correctly classified using the discriminant analysis (see Figure 3). The sPLS-DA explained 41% of the total variance, with vaccenic and behenic acids being the major contributors to variance on loading 1 (Figure 3b). On loading 2, both arachidic acid and EPA were the most contributing fatty acids to the variance (Figure 3c).

**Figure 4:**
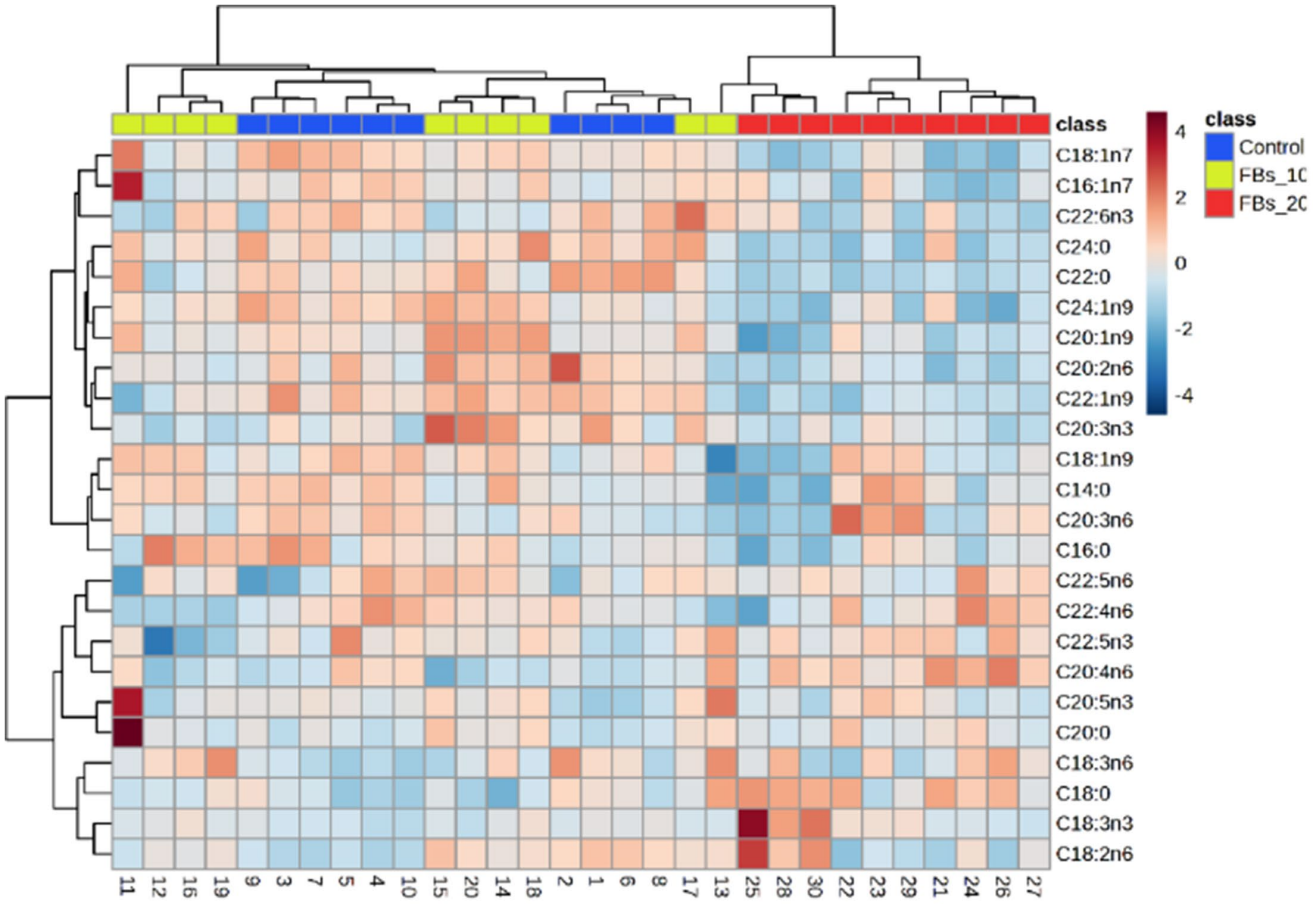
Two-way cluster analysis plot based on all fatty acids of the spleen total phospholipid.

The cluster analysis results, as illustrated in Figure 4, revealed a distinct classification based on the spleen total phospholipid fatty acid composition from the group fed with a 20 mg FBs/kg diet compared to both the control group and the 15 mg FBs/kg diet group. The group with the highest dose of FBs showed a clear separation with zero misclassified cases, highlighting the unique impact of this mycotoxin level in comparison to the other groups.

### 3.5 Antioxidants and lipid peroxidation biomarkers

The assessment of antioxidant parameters [namely the glutathione (GSH) and glutathione peroxidase (GPx)] and the lipid peroxidation biomarker malondialdehyde (MDA) has revealed tissue-specific variations, as shown in Table 6. Meanwhile, no alterations were detected in either the kidney nor the spleen; in contrast, the liver manifested notable modifications. Nevertheless, these modifications were incongruous between the groups fed 10 mg FBs/kg and 20 mg FBs/kg. The group receiving 10 mg FBs/kg feed showed an increase in both the amount of GSH and the activity of GPx. In contrast, the highest dose of FBs (20 mg/kg diet) resulted in a contrasting effect, with a decrease in these antioxidant parameters. In both the kidney and spleen, no significant differences were detected in GSH, GPx, or MDA across the different treatment groups.

**Table 6:** Antioxidant parameters and lipid peroxidation biomarkers in different organs from each treatment (*n* = 10/group).

Parameter	Control	10 mg FBs/kg diet	20 mg FBs/kg diet
	Mean ± SD	Mean ± SD	Mean ± SD
Kidney			
GSH (μmol/g protein)	5.23 ± 1.71	4.59 ± 0.34	3.95 ± 0.79
GPx (U/g protein)	4.51 ± 0.54	4.44 ± 0.50	4.69 ± 1.23
MDA (nmol/g)	89.4 ± 18.4	84.8 ± 16.6	85.4 ± 30.3
Liver			
GSH (μmol/g protein)	5.03 ± 1.64ab	5.72 ± 1.32a	3.94 ± 1.49b
GPx (U/g protein)	4.19 ± 1.48ab	4.68 ± 0.93a	2.88 ± 1.39b
MDA (nmol/g)	61.5 ± 6.53	54.6 ± 8.69	62.4 ± 9.89
Spleen			
GSH (μmol/g protein)	3.45 ± 1.16	5.08 ± 2.46	3.66 ± 0.82
GPx (U/g protein)	2.52 ± 1.01	3.91 ± 2.35	3.18 ± 0.93
MDA (nmol/g)	88.9 ± 26.4	130.9 ± 62.2	91.3 ± 18.1

Data represent mean ± standard deviation (SD). GSH, glutathione; GPx, glutathione peroxidase; MDA, malondialdehyde. a and b letters indicate intergroup differences with *p*-value < 0.05.

### 3.6 Clinical chemistry

The study’s outcomes, detailed in Table 7, highlight significant alterations in serum clinical metabolites. Notably, only creatinine levels among all nitrogenous metabolites showed a significant elevation in animals fed 20 mg FBs/kg compared to the control group. Lipid metabolites also exhibited proportional increases, with total cholesterol and its high-density lipoprotein (HDL) fraction elevating in the group receiving the highest dose of FBs. Among enzyme activities, only gamma-glutamyl transferase (GGT) was affected, showing increased activity in animals fed a high dose of FBs. Ion concentrations remained unchanged regardless of the treatment administered.

**Table 7:** Serum clinical chemistry of experimental rabbits (*n* = 10/group) after 65 days of exposure period.

Component	Control	10 mg FBs/kg diet	20 mg FBs/kg diet
	Mean ± SD	Mean ± SD	Mean ± SD
Total protein (g/L)	61.2 ± 2.51	63.9 ± 3.02	63.8 ± 4.32
Albumin (g/L)	55.8 ± 4.35	54.4 ± 3.50	54.6 ± 3.97
Urea (mmol/L)	5.63 ± 0.57	5.82 ± 0.97	5.35 ± 0.57
Uric acid (μmol/L)	5.00 ± 4.40	3.70 ± 2.83	3.90 ± 2.69
Creatinine (μmol/L)	87.5 ± 8.95b	89.5 ± 10.9ab	99.6 ± 7.18a
Triglyceride (mmol/L)	1.39 ± 0.77	1.66 ± 0.92	0.91 ± 0.29
Total Chol. (mmol/L)	0.60 ± 0.24b	0.70 ± 0.23ab	0.91 ± 0.21a
HDL Chol. (mmol/L)	0.22 ± 0.12b	0.26 ± 0.14b	0.53 ± 0.16a
LDL Chol. (mmol/L)	0.26 ± 0.31	0.31 ± 0.39	0.03 ± 0.18
ALP (IU/L)	45.3 ± 13.3	43.7 ± 18.2	43.3 ± 15.2
AST (IU/L)	24.8 ± 6.76	31.9 ± 19.0	30.5 ± 9.53
ALT (IU/L)	31.2 ± 9.78	37.7 ± 8.21	39.9 ± 11.6
GGT (IU/L)	8.30 ± 3.47ab	5.20 ± 6.88b	11.10 ± 2.51a
LDH (IU/L)	721.2 ± 263.9	892.6 ± 560.3	896.3 ± 480.0
Lipase (IU/L)	312.9 ± 93.8	344.3 ± 154.8	377.3 ± 139.3
CK (IU/L)	1694.1 ± 286.4	2475.7 ± 1103.6	2276.7 ± 642.5
Na (mmol/L)	143.9 ± 2.89	143.5 ± 2.27	143.3 ± 2.98
K (mmol/L)	4.63 ± 0.49	4.67 ± 0.50	4.97 ± 0.60
Ca (mmol/L)	3.93 ± 0.08	3.93 ± 0.27	3.88 ± 0.15
Cor. Ca (mmol/L)	2.34 ± 0.10	2.37 ± 0.23	2.32 ± 0.16
P (mmol/L)	1.24 ± 0.11	1.29 ± 0.21	1.29 ± 0.15
Cl (mmol/L)	97.5 ± 1.51	95.8 ± 2.04	97.5 ± 1.96
Mg (mmol/L)	1.28 ± 0.07	1.32 ± 0.19	1.33 ± 0.13
Fe (μmol/L)	40.5 ± 5.70	38.4 ± 7.02	35.4 ± 7.10

Data represent mean ± standard deviation (SD). ALP, alkaline phosphatase; ALT, alanine transaminase; AST, aspartate transaminase; Chol., cholesterol; CK, creatine kinase; GGT, gamma-glutamyl transferase; HDL, high-density lipoprotein; LDH, lactate dehydrogenase; LDL, low-density lipoprotein. a and b letters indicate intergroup differences with *p*-value < 0.05.

### 3.7 Histopathology

**Figure 5:**
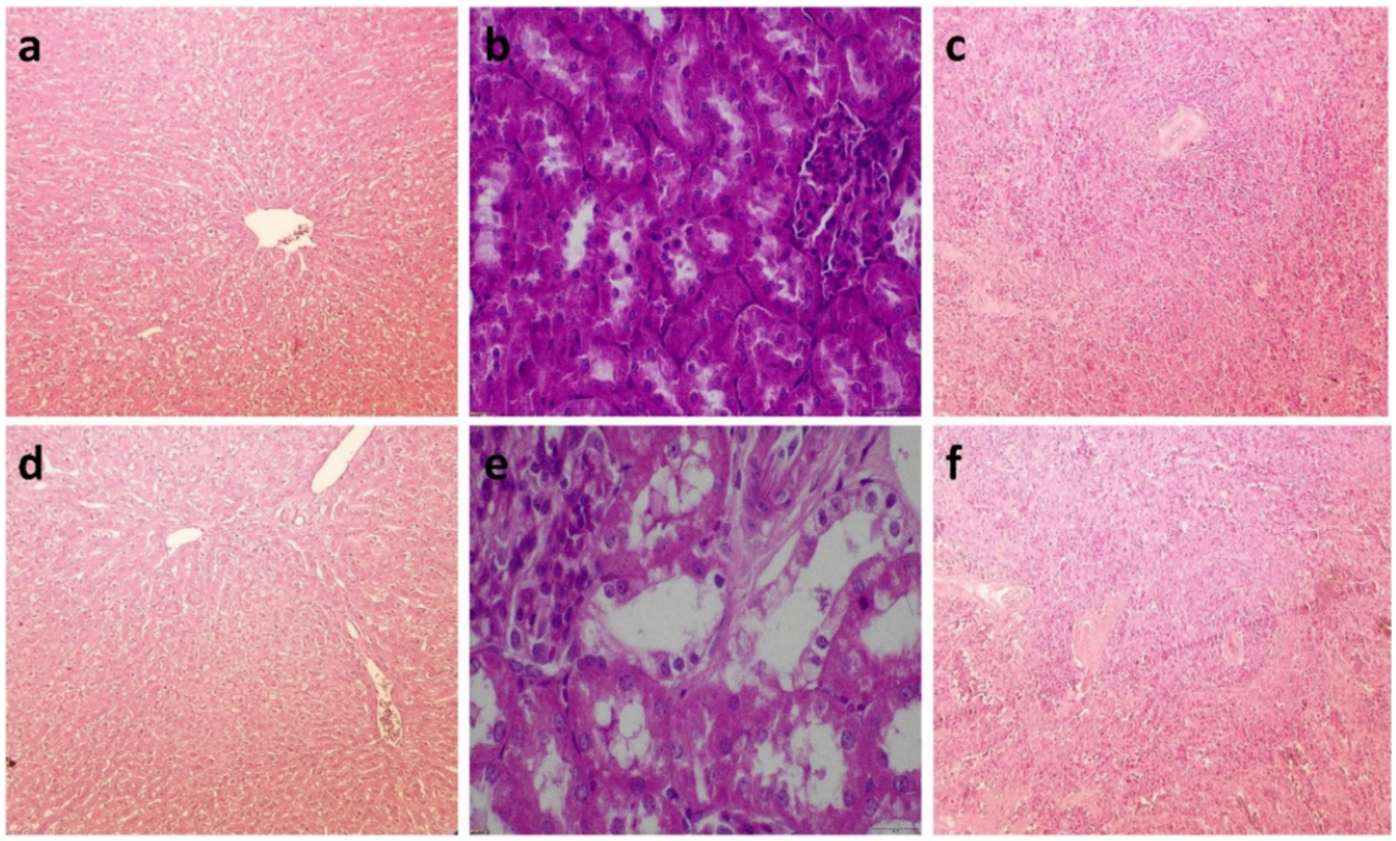
**(a)** Liver of a healthy rabbit, stained with hematoxylin-eosin (H.-E.) at 10X. The cytoplasm of hepatocytes appears vacuolated due to high glycogen content, resulting in a faint stain. **(b)** Kidney of a healthy rabbit from control (H.-E., 40X), whereby the tubules are intact. **(c)** Healthy rabbit spleen (H.-E., 10X), with the Malpighian body containing numerous lymphocytes and lymphoblasts. **(d)** Liver of a rabbit fed 20 mg FBs/kg for 65 days (H.-E., 10X), whereby the glycogen content and vacuolization in the hepatocytes are reduced. **(e)** Rabbit kidney exposed to 20 mg FBs/kg diet (H.-E., 40X), whereby moderate dilation of renal tubules is observed. **(f)** Rabbit spleen after 65 days of 20 mg FBs/kg feeding (H.-E., 10X), where the Malpighian bodies exhibit a narrower ring of lymphocytes.

Throughout the trial, no mortality was observed, and all animals survived until day 65. Table 8 presents the average scores for pathological lesions across various organs. The highest mycotoxin dose resulted in a significant increase in lesion scores compared to the control and 10 mg FBs/kg groups, whereby no lesions were detected. Remarkably, all animals in the 20 mg FBs/kg group exhibited lesions, indicating a 100% pathological finding rate. The solitary hepatocyte necrosis in the liver, tubule epithelial detachment in the kidney, and lymphocyte depletion in the spleen were the most prevalent histological lesions observed. These lesions’ extent of severity varied across organs, whereby mild degrees were observed in the liver and kidneys, while mild to moderate degrees were observed in the spleen (Figure 5).

**Table 8:** Average lesion scores of various organs obtained from experimental rabbits (*n* = 10/group).

Tissue	Control	10 mg FBs/kg diet	20 mg FBs/kg diet
	Mean ± SD	Mean ± SD	Mean ± SD
Liver	0.00 ± 0.00b	0.00 ± 0.00b	1.00 ± 0.00a
Kidney	0.00 ± 0.00b	0.00 ± 0.00b	1.00 ± 0.00a
Spleen	0.00 ± 0.00b	0.00 ± 0.00b	1.50 ± 0.53a

Data represents mean ± standard deviation (SD). a and b letters indicate intergroup differences with *p*-value < 0.05.

#### 3.7.1 Interrelationship between total lesion scores of organs and their relevant parameters

**Figure 6:**
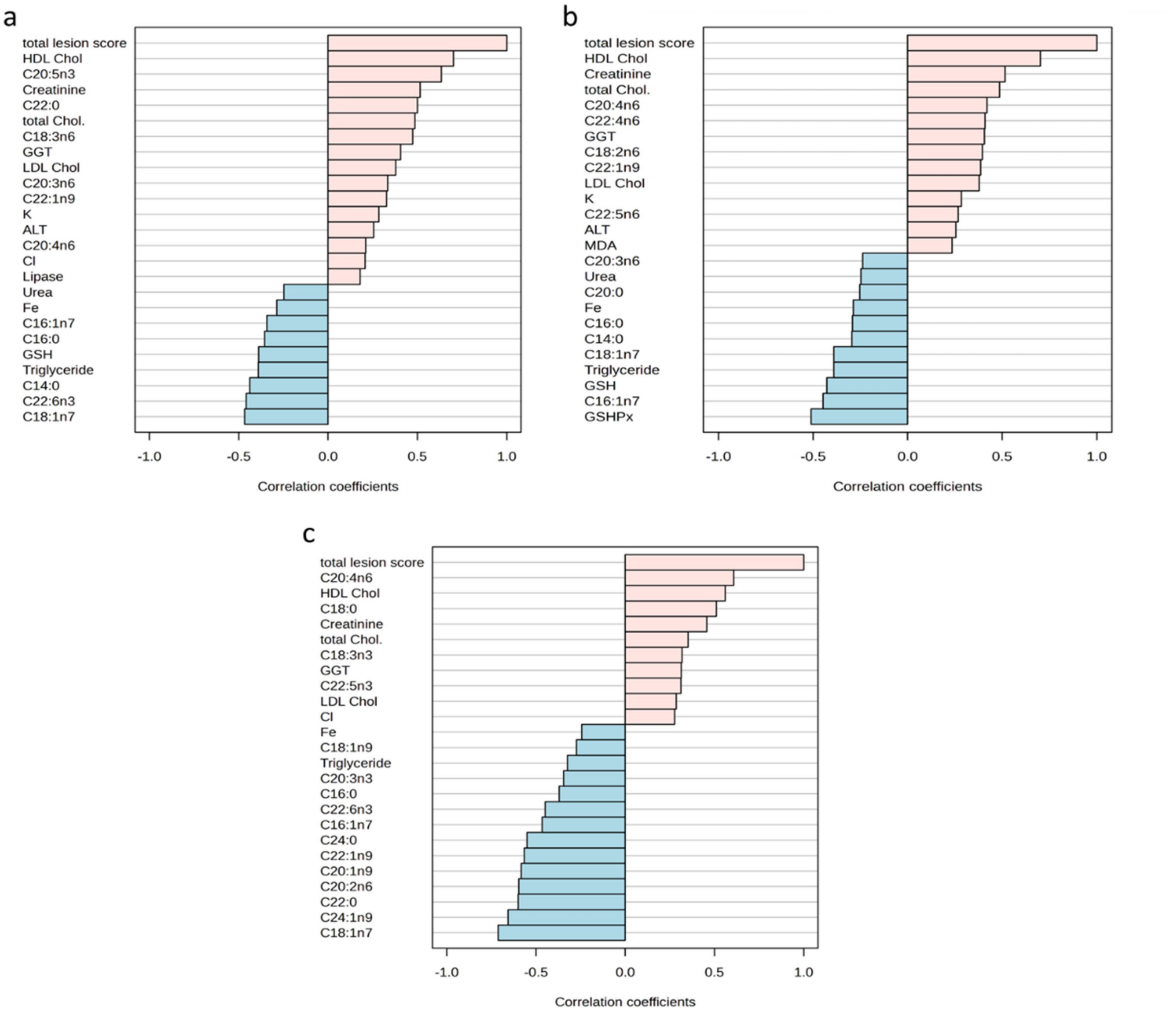
**(a)** Top 25 determined parameters correlated with the total lesion scores of kidneys. **(b)** Top 25 determined parameters correlated with the total lesion scores of livers. **(c)** Top 25 determined parameters correlated with the total lesion scores of spleens. ALT, alanine transaminase; Chol., cholesterol; GGT, gamma-glutamyl transferase; GSH, glutathione; HDL, high-density lipoprotein; LDL, low-density lipoprotein; MDA, malondialdehyde.

Figure 6 illustrates the correlation coefficient between total lesion scores from each tissue and their relevant measured parameters. Notably, the HDL and EPA displayed two positive correlations with the renal total histological lesion scores, providing correlation coefficients of 0.701 and 0.635, respectively (see Figure 6a). A similar positive association for HDL was observed in the liver, also reaching *r* = 0.701. No negative correlations above −0.6 have been found with the kidney or liver total lesion scores with any of the biological variables. Concerning the spleen, the total lesion scores were positively associated with AA (*r* = 0.608), while showing negative correlations with vaccenic acid (*r* = −0.713) and nervonic acid (*r* = −0.657).

### 3.8 Interrelationship between fumonisins dose and all measured parameters

**Figure 7:**
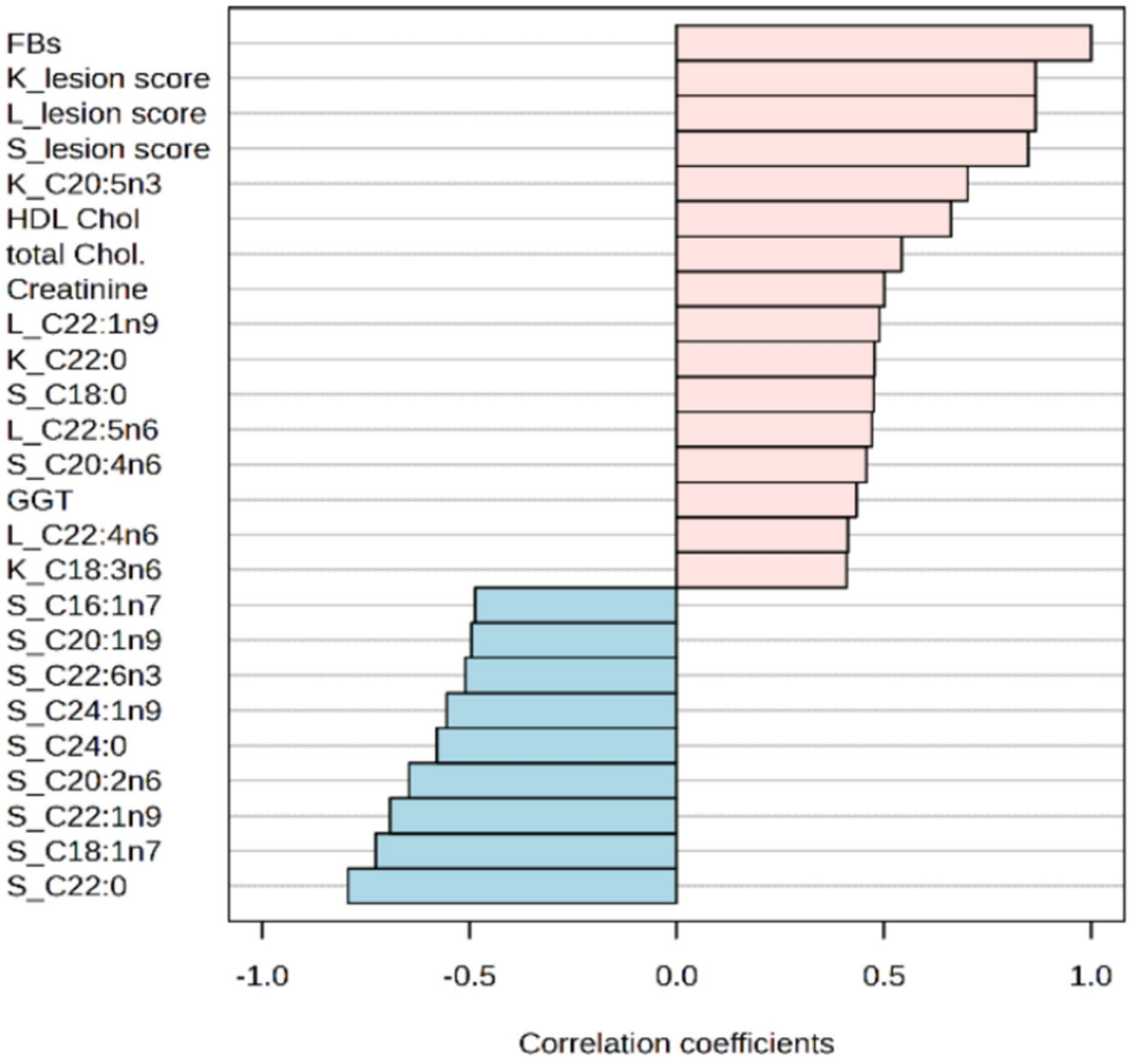
Top 25 correlations between administered doses of FBs and various investigated parameters. Chol., cholesterol; GGT, gamma-glutamyl transferase; HDL, high density lipoprotein; K, Kidney; L, liver; S, spleen.

When investigating the association between mycotoxin doses and various determined parameters, only a few associations yielded values above 0.6 or less than −0.6 (see Figure 7). Notably, average lesion scores for kidneys, liver and spleen showed the highest positive associations, with correlation coefficients of 0.866, 0.866, and 0.848, respectively. In the phospholipids, fatty acids in different tissues exhibited both positive and negative associations with the administered doses of FBs. Specifically, proportions of behenic, vaccenic, erucic and eicosadienoic acids in rabbits’ spleens displayed negative correlations (with *r*-values less than −0.6) with FBs, while the renal EPA proportions showed a positive association (with *r*-value above 0.6). Among all determined serum metabolites, the concentrations of HDL cholesterols were significantly associated with administered mycotoxin doses, providing *r* = 0.662 with a *p*-value below 0.001. In addition, this fraction provided a marked increase, with a fold change above 2-fold when the highest dose of FBs was administered.

## 4 Discussion

### 4.1 Effects of FBs on the growth, organ weights, and feed intake

Generally, the natural rabbit feed intake approach may be a factor contributing to mycotoxin toxicity, as caecotrophy includes the re-ingestion of contaminated excreta; however, this is debatable as FBs may undergo biotransformation to produce less-toxic structures, such as hydrolyzed FB_1_. In this study, animals exhibited the same growth performance, whereby no substantial modifications were detected in body weights or feed intake. This observation is similar to earlier reports on rabbit bucks, when 10 mg FBs/kg feed for a relatively longer period (175 or 196 days) did not alter growth performance (34, 35). Similar findings have also been reported at relatively short periods of exposure (40-, 28-, or 14-days) with comparable doses of 10 and 15 mg FBs/kg feed (11, 13, 20, 36). The dose level appears to have a role in observations, as Ewuola et al. (37) reported that higher levels of FBs compared to ours (24.56 mg/kg) for 35 days had markedly reduced dry matter intake in rabbits, but levels within the range of our dose settings (12.30 mg/kg) did not have a significant impact. In addition, the reported alterations in body weight as well as feed intake in the literature are likely an earlier response phenomenon that had been alleviated in a relatively long timeframe, indicating adaptability to FBs exposure. This corroborates the findings in rabbits, whereby alterations in body weight and feed intake have been recorded within the 1st week of exposure, but no significant difference has been identified after the 2nd week (11). In the present study, bucks appear somehow tolerant to FBs exposure (dose and exposure length), indirectly indicated via the non-elicited alterations in body weight and feed intake.

The absence of alterations in the liver and kidney weights from the 10 mg FBs/kg feed group in this study is consistent with those observed at 10 mg FB_1_ or FBs/kg diet for 14 or 28 days (11, 13, 20, 36). However, a relatively prolonged exposure may provide distinct marked observations, since Ewuola (34) reported that 10 mg FBs/kg feed for a long duration of 196 days did to a marked extent alter the rabbit organ weights. In the present study, the observed high liver and kidney weights at the 10 mg FBs/kg feed compared to the 20 mg FBs/kg exposure level indicate a dose-biphasic response. The observed increase in liver and kidney weights at the lower FBs dose may refer to an adaptive response to elevated metabolic demands associated with detoxification processes. However, at higher doses, the toxic effects may compromise the organ’s capacity to function, leading to cellular damage and a subsequent decrease in organ weight. The decrease in organ weights at higher FBs levels is conceivably a result of necrosis and loss of functional tissue (which are confirmed in this study), as alluded to by the histopathological findings that implicate severe proximal tubular necrosis in the kidneys and mild necrosis in the liver (10, 34). Unlike the liver and kidney, the spleen weight remained unaltered across the different FBs offered levels, suggesting tissue-specific differential sensitivity to FBs-induced toxicity. The spleen’s resistance is likely a consequence of its lower metabolic activity with relation to FBs, as compared to the liver and kidneys, that are primary sites for detoxification, metabolism, and excretion of xenobiotics.

### 4.2 Effects of FBs on the membrane fatty acid composition of organs

Despite several studies employing the membrane fatty acid composition as an endpoint to assess the FBs toxicity in tissues from different animal species, rabbits were slightly investigated, with only three studies focused on the liver, erythrocytes, spermium and testis (6). Hence, the available literature lacks data on the kidney and spleen, which the present paper aimed to investigate. The present study provided various membrane lipidic alterations with various modification degrees across the investigated tissues. The kidney and spleen exhibited the most significant fatty acid responses, especially to the highest dose of FBs, as displayed with the well factorial classification in Figures 2–4; meanwhile, the liver showed a slight extent of modifications. Notably, some alterations, especially in the hepatic tissue, exhibited somehow non-systematic dose response patterns, primarily in proportions of myristic, palmitoleic, vaccenic, arachidonic, erucic acids, as well as the total values of polyunsaturation, the sum of n6 fatty acids, the unsaturation index, and the average chain length. These findings may reflect the organ counter-regulation processes, its homeostatic capacity, or mycotoxin potential in activating specific metabolic events. However, identifying the exact factors/events is likely challenging in *in vivo* models due to the immense number of events that are occurring and potentially interacting at the same time, which may lead to variability in findings compared to *in vitro* studies. Such observations can be seen in the studies of the Gelderblom research group, when *in vitro* (1996) (38) and *in vivo* (1997) (39) studies revealed inconsistency in hepatocellular membrane alterations. Notably, the liver total phospholipid in this study exhibited marked elevations in erucic acid proportion and unsaturation index. In the study of Szabó et al. (20), these fatty acids remained unaltered in rabbit liver membranes upon a 20 mg FB_1_/kg diet for 4 weeks, highlighting the potential differential response due to exposure periods. It is likely that the elevation of erucic acid proportion was a protective mechanism, as research indicates that this acid and other monounsaturated fatty acids generally exhibit protective properties against cytotoxicity, especially in cancerous cell lines (40).

Modifications induced by the FBs exposure were also detected in the kidney and spleen. In the kidney, there were decreases in the proportions of myristic acid and DHA, whereas the sum of n3 fatty acids increased, along with increases in EPA and behenic acid proportions. These findings suggest a disruption in events related to lipid metabolism, which can be mainly attributed to nephrotoxicity without oxidative stress (as the level of lipid peroxidation biomarker remained unchanged across groups) or the kidney’s role in eliminating FBs. For instance, FBs have been reported to interfere with activities of enzymes involved in lipid metabolism (such as elongation and desaturation) (15, 40), which probably emerges indirectly from the potential endoplasmic reticulum stress modulated via the phosphorylation of the protein kinase c-Jun N-terminal kinase (P-JNK) (6). Among altered fatty acids in renal tissue, the proportional decrease of DHA is concerning, as this fatty acid is a vital element for cellular membrane integrity and activity (41, 42). This DHA decrease was probably compensated with the proportional elevation of EPA (consequently increasing the total n3 level), as this fatty acid has been proven to play a remarkable role in anti-inflammatory responses (43). This proposal seems feasible, as EPA elevation was positively associated (*r* = 0.702) with the FBs exposure, likely contributing to function stabilization. In this regard, neither the calculated unsaturation index nor the average chain length varied across the groups, indicating the status of no severe disarrangement of the membrane fatty acid composition.

The study revealed marked alterations in the spleen, whereby monounsaturated fatty acids and their sum were decreased in FBs-treated rabbits, as well as palmitic, eicosadienoic, behenic, docosahexaenoic, lignoceric and nervonic acids. In contrast, the proportions of arachidic and arachidonic acids were increased in rabbits fed FBs, alongside increases in the sum of n6 fatty acids, overall polyunsaturation, unsaturation index and average chain length. These observed modifications were not a consequence of oxidative stress (which was not detected in the spleen); instead, they indirectly suggest alterations in their synthesis, indicating an increased status of membrane permeability (16). This proposal is substantiated by the differing responses noted in mono- and poly-unsaturated fatty acids. The marked increase of arachidonic acid is likely involved in the splanchnic inflammatory and toxicity pathways, demonstrated via its positive relationship (*r* = 0.608) with the total lesion score of the spleen. Apparently, the literature lacks data on spleen membrane lipids in relation to exposure to FBs. Ali et al. (44) reported that exposure to FBs at the EU-recommended level caused slight responses in the total phospholipid fatty acid composition of porcine spleen. Notably, the study by Ali et al. (44) and the current study both examined whole spleen tissues composed of various cell types; thus, an alternative targeting investigation would be more beneficial.

### 4.3 Effect of FBs on the antioxidant and lipid peroxidation markers

Oxidative stress results from an imbalance between the production of reactive oxygen species (ROS) and the amount of low molecular weight antioxidants, and activities of antioxidant enzymes, which is consequently leading to damage of multiple cellular components such as lipids, proteins, and DNA (6, 45). Notably, there is a remarkable gap in the literature regarding the role of oxidative stress in rabbit kidney and spleen toxicities following exposure to FBs; however, the augmentation of oxidative stress by FBs has been reported in numerous *in vivo* studies on different animal species (19, 44, 46, 47), proposing that oxidative stress is more likely a consequence rather than a direct effect of FBs (6, 45). In the present study, regardless of the applied FBs dose, neither the antioxidant biomarkers (GSH and GPx) nor the end product lipid peroxidation marker (MDA) was affected in the kidney and spleen. The spleen is less targeted by FBs exposure, and thus, such findings were anticipated. However, the kidney is generally prone to oxidative damage due to its high metabolic activity (48). When assessing our findings of both antioxidant and oxidation markers alongside datasets obtained from serum clinical metabolites, membrane lipid composition, and histological lesions, it is evident that our results align with earlier reports, proposing that oxidative stress is likely a consequence of various events induced by the FBs toxic effect.

In contrast to the kidney and spleen, the liver exhibited marked decreases in the antioxidant markers (GSH concentration and GPx activity) after 65 days of exposure to 20 mg FBs/kg feed; however, no substantial alteration was detected in MDA levels. These differences suggest the organ-specific response, mainly attributed to the distinct antioxidant capacity, metabolic rate, and physiological functions across organs. It is probable that the liver exhibited an initial hypertrophic adaptation to detoxify FBs, which may be followed by potential organ damage at higher exposure levels. The hepatic findings highlight the initial phase of oxidative stress in hepatocytes that is associated with the initial hypertrophic adaptation, wherein the antioxidant redox system was efficiently utilized in neutralizing the increased ROS levels and diminishing the lipid peroxidation rate. As the hepatic tissue is a primary target for FBs, the typical proposed imbalance in sphingolipid levels (a hallmark of FBs exposure) has contributed to hepatotoxicity, including the augmentation of oxidative stress (20, 49). Further investigations implementing different timeframes and determining the transcription of genes encoding the proteins of the redox-sensitive signal transmission pathways and antioxidant enzymes would provide a better understanding of the hepatic antioxidant capacity to mitigate oxidative stress at various phases of exposure.

### 4.4 Effects of FBs on the serum chemical parameters

The clinical chemistry results revealed that total serum proteins, as well as uric acid, were not substantially altered by FBs exposure. These studies somehow referred to that liver functionality was not compromised during the study period, at least not at a level that compromises protein synthesis. Notably, FBs have been suggested to impair the serum protein production in rabbits (11, 36, 37), probably associated with FBs adverse effects on the digestive system, nutrient bioavailability, and protein synthesis. These studies are typically characterized by a relatively shorter exposure period than ours, in which their settings ranged between 14 and 35 days, suggesting the possible role of exposure duration on these parameters. In a study with 35 days of exposure, even at a higher dose did not compromise the serum protein levels (37), which indicates the probable liver adaptability to the FBs exposure. It is also worth mentioning that the rabbit age may also play a substantial role in sensitivity to FBs, as serum proteins were altered in growing rabbits (which is not the same case in this study) upon exposure to 10 mg FB_1_/kg diet for 84 days (50). However, creatinine levels were increased in the group exposed to 20 mg FBs/kg feed, indicating potential renal impairment. Elevated creatinine levels are a marker of reduced kidney function and have been observed in growing and adult male rabbits (11, 36, 50). This increase in creatinine level is probably due to the nephrotoxic effects of FBs, which cause damage to the renal tubules and impair kidney function (10). Anyways, despite alterations in creatinine being detected, levels were still within the normal physiological range (44–229 μmol/L), suggesting the initial phase of nephrotoxicity. In support, serum ion levels (Na, K, Cl, Ca, P, Fe, and Mg were unaffected by FBs treatments) in this study refer directly to electrolyte balance and normal mineral metabolism, as well as, consequently, the absence of severe nephrotoxicity that affects the homeostatic mechanisms regulating the concentration of these ions.

Serum triglycerides and serum LDL levels remained unchanged, but there was an increase in serum cholesterol and HDL from bucks fed on a 20 mg FBs/kg diet. The hypercholesteremic effect of FBs is commonly documented in animal species, which have also been reported to be in a concentration-related manner in rabbits fed on diets containing 5.0, 7.5, and 10.0 mg FBs/kg for 210 days (51). Alterations in lipids might be due to altered events that interact with cholesterol [such as the disruption of lipid metabolism proposed by Szabó et al. (13)] and events that involve/regulate cholesterol production [including alteration in the nuclear factor LXR expression (52) and/or expression of ABCA1 (53)]. Increased levels of HDL can be rationalized as an adaptive reaction due to disrupting sphingolipid metabolism upon exposure to FBs, implying distortion in lipid homeostasis. According to Vaidya et al. (54), sphingosine-1-phosphate is known to have a modulating effect on cholesterol efflux and HDL metabolism. Given the alteration in total cholesterol and HDL concentrations, the determined steady level of LDL was not anticipated and probably indicates a selective effect of FBs on different components of the lipid profile. In this study, serum lipid findings (especially with HDL providing a correlation (*r* = 0.701) with the liver total lesion score), combined with serum GGT findings, propose the presence of hepatotoxicity; however, the liver functionality was not severely compromised as protein levels were not altered and the altered levels fell into the normal physiological ranges.

Except for the activity of GGT, which increased markedly in rabbits receiving 20 mg FBs/kg diet, serum enzyme activities (ALP, AST, ALT, LDH, CK, and lipase) were not significantly changed. The majority of studies on FBs exposure in rabbits have experienced elevated levels of ALP, AST, ALT, LDH, and GGT activities, while controversial findings were obtained with lipase and CK activities (10, 11, 50, 51, 55). Notably, these cited reports were recorded at a relatively short duration of exposure (5, 14, 21, and 28 days), in growing rabbits for a relatively prolonged exposure (84 and 210 days), and/or at an extremely high level of FBs (630 mg FB_1_/kg diet), which are not the case in this study. Thus, compared with available literature, our findings indirectly propose the rabbit’s liver and kidney potential capacities for adaptability to FBs exposure during a timeframe and the differential sensitivity depending on the age and dose level. Typically, these enzymes are regarded as biomarkers for liver and kidney functionality, and thus, alterations in their activities are attributed to hepatotoxicity and nephrotoxicity events caused by FBs exposure. Within this context, elevated GGT levels, along with elevated creatinine levels in this study, may serve as an indicator of liver and kidney stress or damage, whereas the lack of substantial modifications in other enzyme activities marks that the hepatotoxicity and nephrotoxicity are slight/mild, which has been confirmed by histopathological assessment.

### 4.5 Effects of FBs on histological lesions

The histological results of this study confirm earlier works wherein the primary target organs of FBs were the liver and kidneys, with the spleen being a less affected organ (10, 34, 36). For illustration, Gumprecht et al. (10) observed that there was nephrotoxicity (necrosis of the proximal tubular and dilated tubules) and hepatotoxicity (the presence of vacuoles in hepatocytes and stasis of bile) in rabbits that were intravenously exposed to FB_1_, with renal lesions being more severe. In our study, both the liver and kidney average lesion scores were statistically the same (mild toxicity) at the highest FBs exposure; however, alterations in the membrane fatty acid composition may indirectly reflect the higher toxicity level on the kidney compared to the liver. Notably, both liver and kidney weight decreased with the highest FBs exposure level, corroborating histopathological findings that were attributed primarily to the disruption of sphingolipid metabolism. However, the observed moderate dilation in renal tubules with partial preservation of tissue architecture and no signs of fibrosis is likely pointing out the intact integrity of the tubular basement membrane and the potential tissue regeneration (56). FBs exert their toxic effects primarily through the disruption of sphingolipid metabolism and, consequently, affect cellular integrity and activity. For instance, FBs have been reported to induce apoptosis in hepatocytes and nephrocytes in rodents (57). As FBs provided a high association with lesion scores (see Figure 7), this consequent event likely underlies the hepatocyte necrosis observed in the liver and the epithelial detachment in the kidney tubules. Furthermore, disruption in membrane lipids likely contributes to nephrotoxicity, such as the observed association (*r* = 0.635) between renal total lesion score and EPA. A similar approach can be established for the spleen, as positive and negative correlations have been detected between altered membrane fatty acids [namely AA (*r* = 0.608), vaccenic acid (*r* = −0.713), and nervonic acid (*r* = −0.657)] with the spleen total lesion scores. In addition, the slight increase in spleen lymphocytes in rabbits that were on a diet consisting of 20 mg FB_1_/kg may be justified in terms of immunotoxicity connected with FBs, although the globulin concentrations were steady across treatments. It has been documented that FBs are potential immunotoxicants capable of interfering with immune function through modulation of cytokine secretion and lymphocyte proliferation in mice (58), rats (59, 60), pigs (61) and chickens (62).

## 5 Conclusion

The study emphasized the complex dose-dependent effects of FBs on splanchnic organ toxicity in rabbits. Meanwhile, the growth performance remained unaffected; marked changes in weight were observed in the liver and kidneys, whereas the spleen weight remained unaltered. The findings reflect the roles of both the liver and kidney in detoxification and their susceptibility to damage induced by FBs, as well as highlighting the organ-specific nature of FBs toxicity. The organ sensitivity/toxicity to the highest dose of FBs was illustrated through altered histological lesion scores and serum metabolites, while certain bio-components expressed interrelationships with FBs exposure and/or its toxicity. The observed alterations in fatty acid proportions indicate modulation in lipid metabolism as a critical mechanism underlying adaptation to FBs toxicity, demonstrating the value of examining lipid process pathways in the toxicological diagnosis of FBs. Overall, the study outcomes add to our understanding of the detrimental effects of FBs on rabbit health. However, further studies employing varied designs (such as dose and duration of exposure) are crucial for exploring the consequences of FBs in rabbits.

## Data Availability

The original contributions presented in the study are included in the article/supplementary material, further inquiries can be directed to the corresponding author.

## Footnotes

^1^ https://arriveguidelines.org
